# The diagnostic value of PIVKA‐II, AFP, AFP‐L3, CEA, and their combinations in primary and metastatic hepatocellular carcinoma

**DOI:** 10.1002/jcla.23158

**Published:** 2019-12-10

**Authors:** Famei Qi, Aihua Zhou, Li Yan, Xiumei Yuan, Danni Wang, Ruoyun Chang, Yujun Zhang, Funa Shi, Xiaomei Han, Jinxia Hou, Lianhua Wei, Xu Zhang

**Affiliations:** ^1^ Department of Clinical Laboratory Gansu Provincial Hospital Lanzhou China; ^2^ Key Laboratory of Molecular Diagnostics and Precision Medicine for Surgical Oncology in Gansu Province Gansu Provincial Hospital Lanzhou China; ^3^ Jiangsu Key Laboratory of Medical Science and Laboratory Medicine School of Medicine Jiangsu University Zhenjiang China

**Keywords:** AFP, AFP‐L3, CEA, hepatocellular carcinoma, PIVKA‐II

## Abstract

**Background:**

Early diagnosis decreases the mortality of hepatocellular carcinoma (HCC). We aimed to investigate the usefulness of PIVKA‐II, AFP, AFP‐L3, CEA, and their combinations in the diagnosis of primary and metastatic HCC.

**Methods:**

One hundred and twenty patients with primary HCC (PHC), 115 with metastatic HCC (MHC), 89 with chronic liver disease (CLD), and 116 healthy volunteers were included. The diagnostic values of each marker and their combinations for HCC diagnosis were represented by ROC curve analyses.

**Results:**

PIVKA‐II, AFP, and AFP‐L3 levels in PHC group were higher than that in normal control, CLD, and MHC groups. CEA levels in MHC group were higher than that in the other three groups. When the four markers were analyzed individually, PIVKA‐II showed the highest positive rate in PHC group (76.7%) and CEA showed the highest positive rate in MHC group (69.6%). PIVKA‐ II showed the largest area under ROC curve (AUC = 0.835) to discriminate PHC group from CLD group. Combined PIVKA‐II with AFP‐L3 increased the AUC to 0.910. CEA showed the highest AUC (0.849) to discriminate MHC group from CLD group. Combined CEA with PIVKA‐II increased the AUC to 0.866. AFP‐L3 alone showed the highest AUC (0.890) to discriminate MHC group from PHC group. Combined PIVKA‐II with AFP‐L3, and CEA increased the AUC to 0.957.

**Conclusion:**

PIVKA‐II, AFP‐L3, AFP, and CEA are effective biomarkers for the diagnosis of PHC and MHC. Their combinations could improve the diagnostic performance compared with each marker used alone in detecting PHC and MHC.

## 1 INTRODUCTION

Hepatocellular carcinoma (HCC) is one of the most common malignancies worldwide.^1^ Every year there are more than 500 000 newly diagnosed HCC cases.^2^ HCC has a variable geographical distribution, and the incidence in developing countries is two to three times higher than that in Western countries. In Eastern Asia and Middle Africa, the age‐adjusted incidence rate ranges from 20 to 28 cases per 105 people in men and around 55% of cases are in China.^3^ However, even in the western countries such as the United States (USA), the incidence of HCC is increasing,^4^ it is predicted to continue to increase in the following ten years.^5^

Hepatocellular carcinoma generally has no clinical symptoms in the early stage, and 2/3 of cases are found in the middle and advanced stage, when only about 20% of patients are suitable for surgical resection.^6^ Vascular invasion or extrahepatic tumor spread lead to no curative treatment options available. Metastatic HCC (MHC) is mainly determined by progression of the underlying liver disease rather than by the extrahepatic metastases.^7^ In China and Africa patients who present with symptoms usually die within 4 months^8^ but longer survival is possible in western countries.^9^ Therefore, screening and early diagnosis are vital to reduce the high mortality of HCC.

Several methods are available for HCC diagnosis. The recommended noninvasive methods include imaging techniques such as magnetic resonance imaging and the use of tumor markers such as AFP. However, in up to 40% of HCC patients, AFP levels are normal, especially in the early stage of the disease, which reflects low sensitivity. In order to improve the clinical outcome of patients, it is necessary to determine more reliable serum biomarkers.

PIVKA‐II is also known as des gamma carboxy prothrombin (DCP) and is an abnormal prothrombin molecule that is generated due to acquired defect in the posttranslational carboxylation of the prothrombin precursor in malignant cells. In 1984, Liebman et al^10^ used a radioimmunoassay to detect serum PIVKA‐II levels in patients with HCC and found that 91% of patients showed a significant increase. This finding has since been successfully applied in clinical practice.^11^ PIVKA‐II combined with Golgi protein 73 (GP73) showed higher accuracy than AFP in early HCC diagnosis.^12^ PIVKA‐II has been included in markers for auxiliary diagnosis of HCC in the *Standardization of Diagnosis and Treatment for Hepatocellular Carcinoma (2011 Edition)* issued by National Health Commission of the People's Republic of China. Prothrombin induced by vitamin K absence‐II (PIVKA‐II) and *Lens culinaris*‐agglutinin‐reactive fraction of AFP (AFP‐L3) have both been approved by FDA for risk stratification but not surveillance of hepatocellular carcinoma in the USA.^13^

Serum carcino‐embryonic antigen (CEA) is a relatively non‐specific antigen used in the clinical diagnosis of gastrointestinal cancer.^14^ The elevated level of CEA is found in some patients with poor HCC prognosis.^15^ Therefore, this marker may be useful for classification of prognosis. Many studies have reported the value of different tumor markers in the diagnosis of HCC, but there is currently no study using PIVKA‐II, AFP, AFP‐L3, CEA, and their combinations for the differential diagnosis of primary and metastatic HCC. Because of the high prevalence of HCC cases in China, seeking a set of diagnostic markers for HCC with better sensitivity and specificity than those in current use is particularly important. The aim of this study was to find an effective method to improve the early detection and accurate diagnosis of HCC, thus benefiting the prognosis of patients.

## 2 MATERIALS AND METHODS

### 2.1 Patients and grouping

Patients with HCC treated in Gansu Provincial Hospital from 2016 to 2018 were included. The inclusion criteria for patients with HCC were as follows: (a) 18‐85 years old; (b) Patients with pathologically confirmed HCC; (c) Patients meeting the Chinese guidelines Standardization of Diagnosis and Treatment for Hepatocellular Carcinoma (2017 Edition) as follows: (a) According to CT, MRI or ultrasound results, typical imaging lesions of HCC are seen, and typical blood flow changes occur in the lesions; (b) CT, MRI, or ultrasound suggest suspected small nodules, which are confirmed by PET examination; (d) Subjects had not received surgery, radiotherapy, chemotherapy, and other treatments. The exclusion criteria for patients and controls were as follows: (a) Subjects with missing laboratory detection data; (b) Subjects with missing clinical and medical history key data; (c) Subjects with severe hemolysis, microbial contamination, or jaundice; (d) Subjects that did not meet the requirements for sample collection or treatment; and (e) Subjects withdrawing from the trial based on the medical consideration by investigators. The included patients were grouped into PHC and MHC groups according to diagnosis. Patients with clinically confirmed MHC, mainly including: (a) Patients with a history of HCC and clinical manifestations of liver tumors; (b) Patients with imaging examination showing a solid liver space‐occupying lesion, most of which were scattered or multiple; and (c) Patients with liver metastasis found during surgery for primary disease, the diagnosis of which needed pathological examination. The patients with non‐viral liver diseases (including autoimmune liver disease, drug‐induced liver injury, and fatty liver) and hepatitis (mainly hepatitis B and hepatitis C) were included in the chronic disease group. The study was approved by the Research Ethics Committee of Gansu Provincial Hospital.

### 2.2 Clinical data collection and examination methods

Three microliters of venous blood was collected from the patients and healthy controls. The serum was separated and stored in −80°C freezer until use. PIVKA‐II, AFP, and CEA levels were measured in microparticle chemiluminescence instrument (Abbott I2000). AFP‐L3 was measured by enzyme‐linked immunosorbent assay (ELISA) (Shanghai Jonln Biotechnology). The cutoff values for the four markers were as follows: PIVKA‐II >39.54 mAu/mL, AFP >8.78 ng/mL, AFP‐L3 >7.26 ng/mL, and CEA >5.0 ng/mL.

### 2.3 Statistical analysis

Data were analyzed using SPSS 25.0 statistical software (IBM Corp.). Kruskal‐Wallis *H* Test was used for the comparison of the marker levels in four groups; the Bonferroni method was used for comparisons between each two groups, and *P* ≤ .05 was considered statistically significant. The diagnostic value of each index for PHC or MHC was represented by receiver operating characteristic (ROC) curve, and these were used to calculate the sensitivity, specificity, Youden index, and other indexes.

## 3 RESULTS

### 3.1 Demographic data of subjects

The baseline measurements for the four groups in this study are shown in Table 1. All of the measurements showed a significant difference between the four groups. Patients in PHC and MHC groups were older than those in CLD group and normal control group. It was reported that primary HCC can occur at any age, with the most patients between 40 and 59 years old and a male‐to‐female ratio of 2‐5:1. About 60% patients were male in chronic liver disease group while about 76% patients were male in PHC group. Thus, there was a slight deviation in patient age. The serum levels of various liver function indicators including ALT, AST, GGT, and ALB in patients with HCC were higher than those with chronic liver disease. The decreases in blood cell indexes including Plt, RBC, and Hb were also observed in patients with HCC.

**Table 1:** Baseline data for the four groups included in the study

	Normal control group (n = 116)	CLD group (n = 89)	PHC group (n = 120)	MHC group (n = 115)	*P*
Age (years)	43 (35‐56)	47 (35‐57)	56 (48‐66)	63 (52‐70)	<.001
Sex (n, %)					
Male	50 (43.1)	54 (60.6)	92 (76.7)	80 (69.6)	<.001
Female	66 (56.9)	35 (39.4)	28 (23.3)	35 (30.4)	
ALT (U/L)	18 (14‐25.75)	32 (21‐48.5)	52.4 (33.25‐101.1)	37.4 (18.7‐85.2)	<.001
AST (U/L)	19 (16‐23)	30 (21‐40)	72.5 (37.4‐143.25)	52.9 (31.05‐100.8)	<.001
GGT (U/L)	17 (14‐31)	29.1 (17.95‐48.9)	104.5 (39.86‐186.12)	218 (45.12‐563.5)	<.001
TBIL (μmol/L)	14.75 (10.82‐19.45)	21.2 (16.2‐29.9)	31.7 (18.6‐57.55)	20.88 (15.2‐35.3)	<.001
DBIL (μmol/L)	4.65 (3‐5.9)	6.5 (5.15‐10.35)	10.79 (7.01‐21.64)	3.5 (2.7‐5.5)	<.001
ALB (g/L)	43.20 (41.5‐45.3)	43.2 (36.65‐45.65)	32.07 (28.53‐38.08)	40.1 (36‐43.5)	<.001
Plt (10^9^/L)	212 (176.25‐248.75)	127 (70.5‐178)	124.24 (72.64‐173.35)	146 (93‐195)	<.001
WBC (10^9^/L)	5.6 (4.9‐6.28)	5 (3.96‐6.3)	4 (2.8‐5.89)	5.1 (3.9‐6.7)	<.001
RBC (10^12^/L)	4.74 (4.47‐5.01)	4.57 (3.93‐5.03)	3.13 (2.64‐3.93)	3.81 (3.16‐4.53)	<.001
Hb (g/L)	146 (135.25‐154.75)	144 (120‐161)	89.15 (76‐123.88)	104 (88‐131)	<.001

Abbreviations: ALB, albumin; ALT, alanine aminotransferase; AST, aspartate aminotransferase; DBIL, direct bilirubin; GGT, gamma‐glutamyl transferase; Hb, hemoglobin; Plt, Platelets; RBC, red blood cells; TBIL, total direct and indirect bilirubin; WBC, white blood cells.

### 3.2 Comparison of the diagnostic values of four biomarkers in different groups

The levels of PIVKA‐II, AFP, and AFP‐L3 in PHC group were significantly higher than those in normal control, CLD, and MHC groups (*P* < .05; Table 2). The levels of CEA in MHC group were significantly higher than those in other three groups. The levels of PIVKA‐II in MHC group were significantly higher than those in normal control and CLD groups. AFP‐L3 and AFP levels in MHC group were significantly higher than those in normal control group (*P* < .05). When the four indexes were analyzed individually (Table 3), PIVKA‐II showed the highest positive rate in PHC group (76.7%) and CEA showed the highest positive rate in MHC group (69.6%). In the combined tests, PIVKA‐II or AFP‐L3, PIVKA‐II or AFP, and AFP or AFP‐L3 increased the positive rate of to 92.5%, 91.7%, and 91.7%, respectively, in PHC group.

**Table 2:** The expression levels of four markers in different groups

Groups	n	PIVKA‐II (mAU/mL)	AFP (ng/mL)	AFP‐L3 (ng/mL)	CEA (ng/mL)
Normal control	116	21.9 (19.58‐24.97)	2.71 (2.14‐3.77)	3.44 (2.33‐5.09)	1.56 (1.02‐1.90)
CLD	89	23.4 (16.01‐39.17)	4.7 (2.81‐7.29)^a^	3.21 (2.0‐5.41)	2.14 (1.3‐3.14)^a^
PHC	120	2000 (43.44‐29771.36) a, b	149.39 (8.01‐2000)^a^, ^b^	11.02 (6.83‐12.25)^a^, ^b^	2.90 (1.93‐4.29)^a^, ^b^
MHC	115	40.19 (27.56‐138.34)^a^, ^b^, ^c^	4.27 (2.56‐10.57)^a^, ^c^	4.32 (3.22‐5.33)^a^, ^c^	10.04 (2.98‐782.40)^a^, ^b^, ^c^
H		192.26	152.93	159.59	174.61
*P*		.000	.000	.000	.000

a vs normal control group.

b vs chronic liver disease group.

c vs PHC group, *P* ≤ .05.

**Table 3:** Comparison of the positive rates of four markers in different groups

Indexes	Normal control （n = 116）	CLD (n = 89)	PHC (n = 120)	MHC (n = 115)	*χ* ^2^	*P*
PIVKA‐II	2 (1.7)	21 (23.6)	92 (76.7)	60 (52.5)	154.856	.000
AFP	0 (0.0)	20 (22.5)	89 (74.2)	37 (32.2)	177.3	.000
AFP‐L3	0 (0.0)	18 (20.2)	89 (74.2)	15 (13.0)	135.250	.000
CEA	0 (0.0)	10 (11.2)	19 (15.8)	80 (69.6)	138.629	.000
PIVKA‐II or AFP	2 (1.7)	21 (46.1)	110 (91.7)	76 (66.1)	115.921	.000
PIVKA‐II or AFP‐L3	2 (1.7)	39 (43.8)	111 (92.5)	70 (60.9)	113.386	.000
AFP or AFP‐L3	0 (0.0)	38 (42.7)	110 (91.7)	49 (42.6)	116.702	.000

### 3.3 Evaluation of the diagnostic values of four biomarkers and their combinations in PHC group

**Figure 1:**
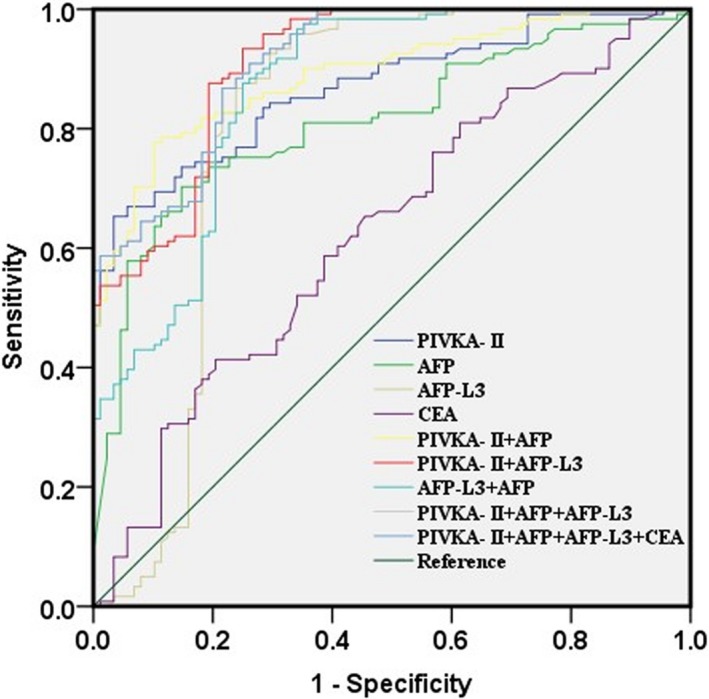
Receiver operating characteristic curves for PIVKA‐2, AFP, AFP‐L3, CEA, and their combinations in PHC group compared to CLD group

The ROC curve analyses of the four markers in PHC group (compared with chronic liver disease group) were shown in Figure 1. When the four biomarkers were analyzed individually, PIVKA‐II, AFP, AFP‐L3, and CEA showed the area under ROC curve (AUC) of 0.835, 0.810, 0.807, and 0.625, respectively (Table 4). PIVKA‐II showed the highest AUC with a sensitivity of 0.835 and a specificity of 0.716. AFP‐L3 showed the highest sensitivity of 0.959 and AFP showed the highest specificity of 0.807. Combined PIVKA‐II with AFP‐L3 increased the AUC to 0.910 with increased sensitivity of 0.876 and specificity of 0.807. The combination of PIVKA‐II with AFP‐L3, and AFP could further increase AUC to 0.914 with an increased sensitivity of 1.000 but a decreased specificity of 0.625. The combination of four markers had no change in AUC (Table 4). These findings indicate that the combination of PIVKA‐II, AFP‐L3, and AFP could improve their abilities to discriminate patients with PHC and chronic liver diseases.

**Table 4:** Receiver operating characteristic curve analyses of the diagnostic values of the four indexes alone and their combinations for distinguishing PHC group from CLD group

Index	AUC	*P*	Cut‐off	Sensitivity	Specificity	Youden index
PIVKA‐II	0.835	.000	33.080	0.835	0.716	0.119
AFP	0.810	.000	11.880	0.736	0.807	0.543
AFP‐L3	0.807	.000	4.367	0.959	0.670	0.629
CEA	0.625	.000	2.520	0.587	0.614	0.201
PIVKA‐II + AFP	0.890	.000	0.343	0.901	0.648	0.549
PIVKA‐II + AFP‐L3	0.910	.000	0.422	0.876	0.807	0.683
AFP‐L3 + AFP	0.865	.000	0.291	0.975	0.648	0.623
PIVKA‐II + AFP‐L3 + AFP	0.914	.000	0.217	1.000	0.625	0.625
PIVKA‐II + AFP+AFP‐L3 + CEA	0.914	.000	0.420	0.868	0.784	0.652

### 3.4 Evaluation of the diagnostic values of four biomarkers and their combinations in MHC group

**Figure 2:**
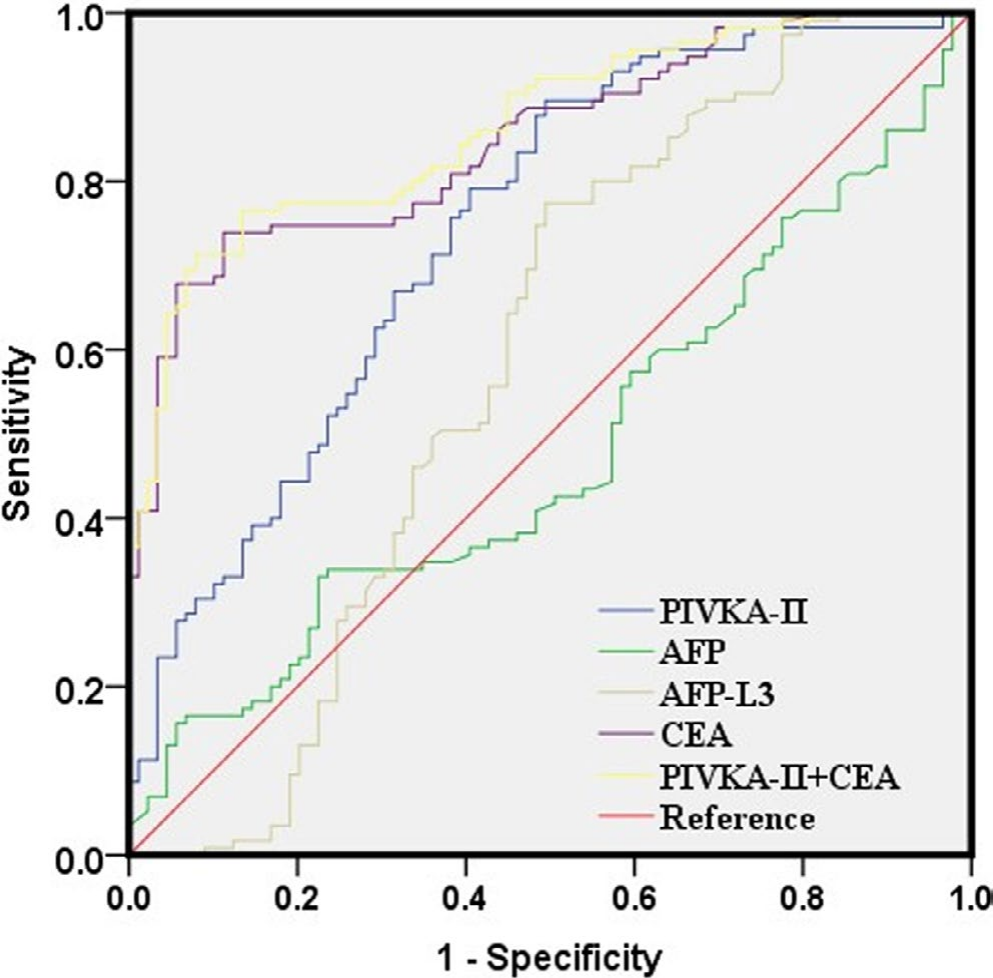
Receiver operating characteristic curves for PIVKA‐2, AFP, AFP‐L3, CEA, and their combinations in MHC group compared to CLD group

The ROC curve analyses of the four markers in PHC group (compared with chronic liver disease group) were shown in Figure 2. When the four biomarkers were analyzed individually, PIVKA‐II, AFP, AFP‐L3, and CEA showed the AUC of 0.744, 0.48, 0.588, and 0.849, respectively (Table 5). CEA showed the highest AUC with a sensitivity of 0.739 and a specificity of 0.888. PIVKA‐II showed the highest sensitivity of 0.896 and CEA showed the highest specificity of 0.888. Combined CEA with PIVKA‐II increased the AUC to 0.866 with increased specificity of 0.933 but decreased sensitivity of 0.696 (Table 5). These findings indicate that CEA, PIVKA‐II, and their combinations could improve their abilities to discriminate patients with MHC and chronic liver diseases.

**Table 5:** Receiver operating characteristic curve analyses of the diagnostic values of the four indexes alone and their combinations for distinguishing MHC group from CLD group

Index	AUC	*P*	Cut‐off	Sensitivity	Specificity	Youden index
PIVKA‐II	0.744	.000	23.405	0.896	0.506	0.402
AFP	0.48	.629	4.385	0.452	0.427	0.121
AFP‐L3	0.588	.032	3.205	0.774	0.494	0.268
CEA	0.849	.000	3.995	0.739	0.888	0.627
PIVKA‐II + CEA	0.866	.000	0.489	0.696	0.933	0.629

### 3.5 Evaluation of the diagnostic values of four biomarkers and their combinations in discriminating PHC and MHC groups

**Figure 3:**
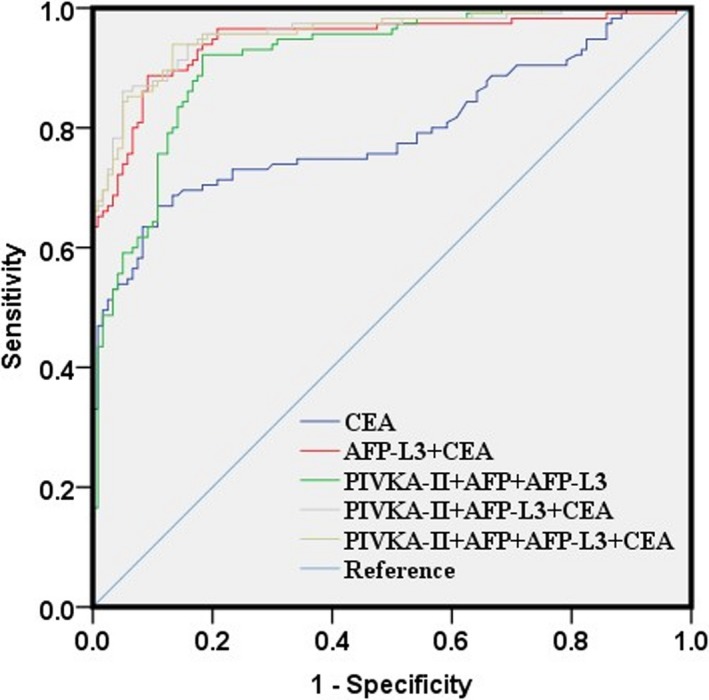
Receiver operating characteristic curves for PIVKA‐2, AFP, AFP‐L3, CEA, and their combinations in MHC group compared to PHC group

The ROC curve analyses of the four markers in MHC group (compared with PHC group) were shown in Figure 3. When the four biomarkers were analyzed individually, PIVKA‐II, AFP, AFP‐L3, and CEA showed the area under ROC curve (AUC) of 0.749, 0.784, 0.89, and 0.795, respectively (Table 6). AFP‐L3 showed the highest AUC with a sensitivity of 0.904 and a specificity of 0.725. AFP‐L3 showed the highest sensitivity of 0.904 and CEA showed the highest specificity of 0.892. Combined PIVKA‐II with AFP‐L3 increased the AUC to 0.917 with increased sensitivity of 0.922 and specificity of 0.792. Combined CEA with AFP‐L3 increased the AUC to 0.945 with increased sensitivity of 0.887 and specificity of 0.908. The combination of PIVKA‐II with AFP‐L3, and CEA could further increase AUC to 0.957 with a sensitivity of 0.861 and a specificity of 0.95 (Table 6). Adding AFP had no alteration in AUC although it increased the sensitivity but decreased the specificity. These findings indicate that the combination of PIVKA‐II, AFP‐L3, and CEA could improve their abilities to discriminate patients with MHC and PHC.

**Table 6:** Receiver operating characteristic curve analyses of the diagnostic values of the four indexes alone and their combinations for distinguishing PHC group and MHC group

Index	AUC	*P*	Cut‐off	Sensitivity	Specificity	Youden index
PIVKA‐ II	0.749	.000	296.110	0.887	0.600	0.487
AFP	0.784	.000	18.105	0.826	0.708	0.534
AFP‐L3	0.89	.000	8.120	0.904	0.725	0.629
CEA	0.795	.000	5.890	0.670	0.892	0.562
PIVKA‐II + AFP	0.805	.000	0.629	0.800	0.725	0.525
PIVKA‐II + AFP‐L3	0.917	.000	0.344	0.922	0.792	0.714
AFP‐L3 + AFP	0.901	.000	0.676	0.817	0.867	0.684
PIVKA‐II + CEA	0.870	.000	0.531	0.661	0.942	0.603
AFP‐L3 + CEA	0.945	.000	0.59	0.887	0.908	0.795
AFP + CEA	0.870	.000	0.518	0.687	0.942	0.629
PIVKA‐II + AFP‐L3 + CEA	0.957	.000	0.674	0.861	0.95	0.811
PIVKA‐II + AFP‐L3 + AFP	0.915	.000	0.400	0.922	0.817	0.739
PIVKA‐II + AFP‐L3 + AFP+CEA	0.957	.000	0.489	0.939	0.867	0.806

## 4 DISCUSSION

The early diagnosis of HCC is essential for curative interventions, which helps improve the prognosis and long‐term survival of patients. The previous studies have shown that PIVKA‐II is helpful for the diagnosis of HCC.^1^, ^16^ Moreover, several studies have shown that AFP‐L3 could improve the detection rate of HCC when it is combined with PIVKA‐II.^17^, ^18^ Lim et al^18^ also suggest that combining AFP, AFP‐L3, and PIVKA‐II improves the diagnostic accuracy for HCC among cirrhotic patients compared with using each marker individually. According to the Guidelines of Japan, AFP, AFP‐L3, and PIVKA‐II are recommended as serological biomarkers in clinical settings and these markers are routinely used to screen for HCC.^19^

In this study, we evaluated the effectiveness of four markers including PIVKA‐II, AFP‐L3, AFP, and CEA in discriminating patients with PHC, MHC, and chronic liver disease. These four markers were analyzed and compared either alone or in combination. As expected, the levels of all four markers were significantly elevated in patients with HCC compared to those with chronic liver disease. PIVKA‐II showed the highest positive rate in PHC group and CEA showed the highest positive rate in MHC group. When these markers were combined, the positive rate of PIVKA‐II/AFP‐L3 in PHC group was increased compared with PIVKA‐II or AFP‐L3 alone. On the contrary, while the positive rate of PIVKA‐II/AFP‐L3 or PIVKA‐II/AFP was lower than CEA alone in MHC group. These data suggest that PIVKA‐II, AFP‐L3, and AFP have complementary effects for the diagnosis of PHC. The reasonable use of these markers can improve the positive rate and accuracy of diagnosis. Moreover, it helps to reduce the rate of missed diagnosis and decrease the rate of misdiagnosis. This is consistent with the results reported in the literature.

PIVKA‐II has been found to be superior to AFP or AFP‐L3 in detecting PHC; this finding is consistent with those from several earlier studies.^20^, ^21^ We also compared the usefulness of AFP, AFP‐L3, and PIVKA‐II both individually and in combination in diagnosing PHC. We found that the combination of PIVKA‐II and AFP‐L3 was the most valuable panel for detecting PHC (AUC 0.910, sensitivity 0.876 specificity 0.800). This result is consistent with that reported in the literature 18, 22 showing that the combination of PIVKA‐II, AFP, or AFP‐L3 has a superior detection of PHC with no significant decrease in specificity in Asian population. Although the combination of three or four markers works better, the cost of examination also increases, which may reduce its potential of use.^21^ Other combinations of two or three markers did not provide superior diagnostic ability. Intriguingly, it has been reported that the changes of PIVKA‐II in PHC are not associated with AFP.^23^ Therefore, PIVKA‐II can improve the positive rate of diagnosis for AFP‐negative patients. The advantages of combined test have also been confirmed in clinical practice. For example, AFP, AFP‐L3, and PIVKA‐II are included as serum biomarkers in the clinical settings of Japan National Health Insurance.^16^ In the future, the sample size will be further expanded, and the association of PIVKA‐II with staging, curative effect and prognosis in PHC will be further studied.

For the diagnosis of metastatic HCC, CEA alone and PIVKA‐II alone showed a better AUC and the combination of CEA and PIVKA‐II showed the highest diagnostic accuracy. However, CEA had a better specificity than PIVA‐II while PIVA‐II had a better sensitivity than CEA in MHC. These findings indicate that PIVKA‐II and CEA has an ability to discriminate patients with MHC and chronic liver disease. CEA is a broad‐spectrum tumor marker that is combined with other biomarkers to diagnose primary HCC.^24^ However, we found that CEA was significantly elevated in MHC, which is helpful for differential diagnosis between primary and metastatic HCC. This is consistent with the findings from Huang et al^25^ showing that the patients with distant metastasis have high CEA levels than those without distant metastases. In addition, AFP‐L3 showed a sensitivity of 0.890 for distinguishing MHC and PHC groups, which was slightly higher than that previously reported in the Chinese population.^26^ The combination of AFP‐L3 and CEA was better to discriminate patients with MHC and PHC. (AUC 0.945, sensitivity 0.887, specificity 0.908). Overall, the sensitivity, specificity, and AUC for combined use were higher than those used alone for distinguishing MHC and PHC groups.

In conclusion, a combination of four biomarkers including AFP, PIVKA‐II, AFP‐L3 and CEA showed better accuracy than either marker alone in distinguishing patients with MHC, PHC, and chronic liver disease. The detection methods are simple, stable and reliable, and thus these markers are suitable for application in hospitals at all levels.

## CONFLICTS OF INTEREST

The authors have no conflicts of interest to be declared.
